# Dynamic Expression and Regulation of Urotensin I and Corticotropin-Releasing Hormone Receptors in Ovary of Olive Flounder *Paralichthys olivaceus*

**DOI:** 10.3389/fphys.2019.01045

**Published:** 2019-08-13

**Authors:** Hong Zhou, Chunmei Ge, Aqin Chen, Weiqun Lu

**Affiliations:** ^1^National Demonstration Center for Experimental Fisheries Science Education, Shanghai Ocean University, Shanghai, China; ^2^Key Laboratory of Exploration and Utilization of Aquatic Genetic Resources, Ministry of Education, Shanghai Ocean University, Shanghai, China; ^3^International Research Center for Marine Biosciences at Shanghai Ocean University, Ministry of Science and Technology, Shanghai, China

**Keywords:** Urotensin I, corticotropin-releasing hormone receptors, *Paralichthys olivaceus*, folliculogenesis, ovarian development

## Abstract

Urotensin I (UI), a fish corticotropin-releasing hormone (CRH) like peptide, has been found throughout vertebrate species that has great effects on adaptive physiology comprising stress-related responses, and osmotic regulation by binding with CRH type I receptor (CRHR1) and CRH type II receptor (CRHR2) in fish. Dynamic expression and regulation of UI and CRH receptors in the olive flounder ovarian follicle were studied so as to make further efforts to understand the role of UI in the development of teleost ovary. The results showed that stage-specific change in UI mRNA levels in ovarian follicles of olive flounder. UI and CRHR1 mRNA levels were higher in stage III follicles (300∼500 μm diameter) compared to stage II (90∼300 μm diameter) and IV (500∼800 μm diameter) follicles, however, the levels of CRHR2 mRNA were decreased in line with the ovarian development from stage II to stage IV. A strong signal of UI protein was observed in the follicular cells and oocyte in stage III and IV follicles by immunohistochemistry. *In vitro* treatment of olive flounder ovarian follicles with human chorionic gonadotropin (hCG) showed that the mRNA expression of UI increased significantly at low concentration and decreased at high concentration at 6 h, but the CRHR1 and CRHR2 mRNA did not change obviously. In addition, the results of incubation with 17α, 20β-dihydroxy-4-oregnen-3-one (DHP) show that UI and CRHR1 mRNA expression were elevated with increasing concentrations at 9 h. All above results indicated that UI and CRH receptors may have a vital effect on olive flounder ovarian development.

## Introduction

Urotensin I (UI) was comprised of 41-amino acid belongs to the superfamily of CRH, which also includes the mammalian UI ortholog, urocortin I (Ucn I) (Lederis et al., 1982; Lovejoy and Balment, 1999; Lu et al., 2004). It was first isolated from the urophysis of the white sucker *Catostomus commersoni* in 1982 (Lederis et al., 1982). Urocortins play a significant role in many crucial functions such as cardiovascular homeostasis, suppression of orexis, and synthesis of glucocorticoid in mammals (Vaughan et al., 1995; Ames et al., 1999; Pittman and Hollenberg, 2009). It also has been demonstrated to be directly participated in regulating steroid production in the ovary (Muramatsu et al., 2001; Florio et al., 2004; Oki and Sasano, 2004; Wypior et al., 2011). Injection of UI irritates pituitary cells of coho salmon *Oncorhynchus kisutch* secreting thyroid-stimulating hormone *in vitro* (Larsen et al., 1998); it also inhibits ingestion and add plasma cortisol content in a dose-dependent pattern in goldfish *Carassius auratus* (Fryer et al., 1983; Bernier, 2006). Moreover, UI plays an important role in adaptive physiology comprising stress-related responses and osmotic regulation in fish (Craig et al., 2005).

It was found that Ucn I mRNA exists not only in the cerebellum and cerebral cortex but also in the pons and hypothalamus of human brain (Takahashi et al., 1998). In human ovary, Ucn I was discovered in intrathecal cells of dominant/non-dominant follicles (including follicles and luteal phase) and atresia follicles (Muramatsu et al., 2001). Ucn I mRNA was not only strongly found in the Edinger-Westphal nucleus (EWN) and olivary nucleus in the rat brain but also found in the front and middle portion of the pituitary (Bittencourt et al., 1999). The expression of UI mRNA was basically detected in cerebellum, hypothalamus, telencephalon/preoptic area, medulla oblongata, and optic tectum/thalamus. Whereas, it was not discovered in pituitary and olfactory bulb in goldfish brain (Bernier et al., 1999). However, the levels UI mRNA was discovered in the nucleus of outboard tuberis and preoptic area (Morley et al., 1991). Even though UI exist in given brain areas in both fish and mammals, a primary origin of UI in the circulation of fish was considered to be the caudal neurosecretory system (CNSS) (Lu et al., 2004). UI mRNA was primarily detected in the urophysis of carp and rainbow trout instead of in the brain, implying that UI has a relatively large effect on peripheral tissues compared with the brain (Lovejoy, 2009). However, the changes of UI in ovarian development has not been explored so far in fish. It was still required further research to demonstrate if UI has a physiological effect on the regulation of reproduction or not.

Since urocortin was one of members of the CRH family, it combines CRH receptors subtypes, CRH type I receptor (CRHR1) and CRH type II receptor (CRHR2), for action on stress-related responses, and reproduction (Makrigiannakis et al., 2018). In mammals, the CRH receptors have been found in the female reproductive system and taken part in domination of the hypothalamic-pituitary-ovarian axis (Makrigiannakis et al., 2018). In fishes, two type of CRH receptors, CRHR1 and CRHR2, have also been cloned and characterized (Arai et al., 2001; Pohl et al., 2001). Furthermore, the high mRNA expression of CRHR1 and CRHR2 have been observed in the ovary of African cichlid fish *Astatotilapia burtoni* and Japanese pufferfish *Fugu rubripes* (Cardoso et al., 2003; Chen and Fernald, 2008). In salmon, CRHR1 was discovered in the ovary, heart, skeletal muscle, gill and brain, but CRHR2 was only found in brain and heart by RT-PCR (Pohl et al., 2001). The demonstration of combined research in chum salmon *Oncorhynchus keta* and catfish *Silurus asotus* indicates that CRHR1 and CRHR2 cannot distinguish CRH and UI (Singh and Rai, 2011). It is still ambiguous whether CRH peptides regulate the ovary development and oocyte maturation by activating CRH receptors.

Previous studies have demonstrated that the expression levels of UI mRNA showed a significant seasonal alteration, meanwhile, the gonadal index (GSI) in April was higher than August to October (Lu et al., 2007; Westring et al., 2008). Moreover, UI from the brain has been shown to play a role in the regulation of cortisol levels in spawning Cherry Salmon *Oncorhynchus masou* (Westring et al., 2008). In present study, our objective was to determine whether UI and CRH receptors are expressed in the olive flounder ovary during ovarian development, and regulated by human chorionic gonadotropin (hCG) or 17α, 20β-dihydroxy-4-oregnen-3-one (DHP). Therefore, we analyzed temporal expression patterns of UI and CRH receptors in olive flounder ovary in the time of the sexual maturity and their stage-dependent in isolated budding follicles. Meanwhile, the localization of UI in olive flounder ovary was detected by immunohistochemistry. In addition, using *in vitro* experiment, we also investigated whether UI and CRH receptors expression were affected by hCG and DHP in olive flounder.

## Materials and Methods

### Animals

Gynogenetic olive flounders were raised in recirculating aquaculture system of seawater (30‰) at the Central Experimental Station of Chinese Academy of Fisheries Sciences (Beidaihe, Hebei, China) (Liu et al., 2012). The test was carried out at the same site in April 2018. A total of 6 gynogenetic olive flounders (weight: 2,000 ± 200 g) were casually assorted in 3 tanks where there was flow-through, filtered seawater (30‰) system at 20 ± 1°C over 2 weeks. Each water tank was surrounded by black plastic light-proof curtains, and was equipped with white fluorescent lamps for artificial lighting. The average light intensity was about 40 lux, which was measured at the bottom of each tank. In order to reduce the influence of feeding, fish were starved throughout the experiment. The experimental protocol was approved by the Institutional Animal Care and Use Committee (IACUC) of Shanghai Ocean University (SHOU), Shanghai, China, and abides by the Guidelines on Ethical Treatment of Experimental Animals established by the Ministry of Science and Technology, China.

### Isolation and Incubation of Olive Flounder Follicles

The six flounders were anesthetized with tricaine methanesulfonate (200 mg/L) and decapitated before anatomy. The ovaries were then dissected and weighed to analyze gonad-somatic index (GSI) [GSI = gonad weight(g)/total body weight(g) × 100]. One part of gonad samples was fixed and used to determine the developmental stage by histological and immunohistochemical analysis (Radonic and Macchi, 2009; Ding et al., 2013). The other part of gonad samples was frozen in liquid nitrogen at once for subsequent analysis of genetic expression.

The ovaries were put in a 100-mm culture dish which contains 60% Leibovitz L-15 (Gibco, Carlsbad, CA, United States) medium. Ovarian follicles were carefully severed with the help of fine forceps and blades. The follicles were measured by ophthalmic micrometer under an anatomical microscope. The healthy follicles at different developmental stages were grouped according to the reference follicles diameter (Radonic and Macchi, 2009; Ding et al., 2013): stage II 90∼300 μm, stage III 300∼500 μm, and stage IV 500∼800 μm. Stage IV follicles with normal shape and clear boundary were chosen and incubated in 24-well culture plates (50 follicles/well) with 60% L-15. The culture plates were incubated at 16°C in an incubator for treatment experiment of recombinant hCG (0, 10, 20, and 50 IU) (Sigma-Aldrich, United States) for 3, 6, and 9 h or DHP (0, 10, 100, and 1000 ng/ml) (Sigma-Aldrich, United States) for 9 h, respectively. There are six replicates for each hCG or DHP concentration treatment. After incubation, the samples were collected and directly frozen in liquid nitrogen for succeeding analysis of genetic expression.

### Quantitative Real-Time RT-PCR (qPCR)

Total RNA was extracted from ovaries and follicles at different stages by RNAiso Plus (TaKaRa, Japan) as recommended by the manufacturer. The purity and integrity of RNA was analyzed by Nano Drop ND-1000 spectrophotometer (Nanodrop Technologies Inc., Wilmington, DE, United States) and 1% agarose gel, respectively. 1 μg total RNA was treated by PrimeScript^TM^ RT reagent kit (Takara, Dalian, China). qPCR was performed using SYBR Premix Ex Taq^TM^ (TaKaRa, Dalian, China) in an ABI 7500 (Applied Biosystems, Carlsbad, CA, United States). Relative quantification of the target gene transcripts was analyzed using β-actin gene expression as the reference gene. Following were the conditions for qPCR: 2 min at 50°C, 10 min at 95°C and then 40 cycles of the following process of 15 s at 95°C and 30 s at 60°C. The primers were designed using Primer Premier 6 software (PREMIER Biosoft International, Palo Alto, CA, United States) and synthesized commercially (Sangon Biotech, Shanghai, China) (Table 1). To determine the specificity of the amplification, the melting curve analysis of the PCR products was performed to ensure that only one fragment was amplified. The 2^–ΔΔ*Ct*^ method was utilized to analyze the real-time PCR data (Livak and Schmittgen, 2001).

**TABLE 1:** Primer sequences used for real-time PCR amplifications.

**Gene**	**Primers (5′–3′)**	**GenBank**
UI	F: GACCTGCTGAGCGACAAC R: TCATCCTCGGCTATCTGG	XM_020105024.1
CRHR1	F: ACCTCATCACCGCCTTCATCC R: AGCAGCCCTCGCCAAACAT	XM_020113112.1
CRHR2	F: TGGCACCGTTGGCAGGACAA R: CGGAGGCTGCGAGGAGATTACA	XM_020102084.1
β-actin	F: GGAAATCGTGCGTGACATTAAG R: CCTCTGGACAACGGAACCTCT	HQ386788.1

### Immunohistochemistry

Paraformaldehyde-fixed ovary were dehydrated in ethanol, cleared in xylene and embedded in paraplast. Every section (10-μm-thick) was cut on a microtome (Leica, Germany) and 10 μm paraffin sections were stained using hematoxylin and eosin to observe the follicle cells at different stages. Briefly, tissue sections were dewaxed in xylene and rehydrated in gradient alcohol. Endogenous peroxidase activity was blocked with 3% H_2_O_2_ in methanol before slides were placed in 0.01M citrate buffer and heated in a water bath for 20 min at 95°C. After cooling, sections were rinsed in PBS. For UI immunohistochemistry, sections were treated with fetal bovine serum (FBS) blocking solution (1% blocking, dissolved in MABT, and 5% FBS in PBST, PBS with 0.1% Triton X-100) for 1 h at room temperature (RT) to reduce non-specific staining and incubated with 1:500 rabbit anti-UI antibody (Lu et al., 2004) diluted with PBS in a moist chamber at 4°C overnight. The moist chamber was transferred into an air oven at 37°C for 45 min, then washed six times at RT in PBST for 15 min each time at RT and 1:500 goat anti-rabbit I gG (H + L) highly cross adsorbed secondary antibody (Thermo Fisher Scientific, United States), diluted with PBS for 1 h in dark. Finally, the sections were stained with the DAB staining kit (Boster, China). The negative control group was incubated with PBS instead of the primary antibody to verify the specificity of immunostaining.

### Statistical Analysis

SPSS 25.0 software package was used for all statistical analyses. For statistical analysis, the expression levels of target genes were standardized to that of the internal control β-actin and expressed as fold change relative to the control group. Statistical differences were detected using one-way ANOVA, and LSD post-mortem analysis was then performed to assess the differences between specific groups. The effects of time, concentration, and their interactions were analyzed using a two-way ANOVA. All the results were expressed by mean ± SEM, and the difference was statistically significant (*p* < 0.05).

## Results

### Determination of Ovarian Development Stage

**FIGURE 1:**
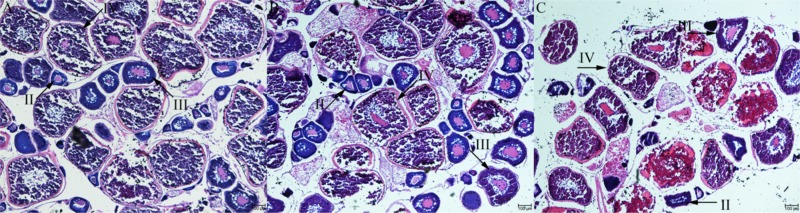
Histological section of Japanese flounder ovaries at different stages of follicle development. stage II **(A)**, stage III **(B)**, and stage IV **(C)**. Scale bar = 100 μm.

The GSI of olive flounders at ovarian stage II, III and IV were increased progressively at 1.54 ± 0.31, 6.03 ± 4.04, and 9.4 ± 2.85, respectively. At the stage III and stage IV ovaries were become mature as vitellogenic follicle of 300–500 μm in diameter and postvitellogenic follicle of 500–800 μm in diameter, respectively. There were some small yolk granules in the mazarine cytoplasm (stage II follicle). The germinal vesicle (GV) was found in the central position and many large lipid droplets and cortical alveoli were dispersed in the cytoplasm (stage III follicle). As the number and size of yolk granules increased, it almost completely occupies the entire cytoplasm (stage IV follicle) (Figures 1A–C). The results of morphology and histology indicate that the ovarian development of olive flounder was asynchronous, and oocytes of different development stages can be simultaneously found in the same ovary (Figure 1).

### Expression of UI and CRH Receptors at the Ovary Level During Ovarian Development

**FIGURE 2:**
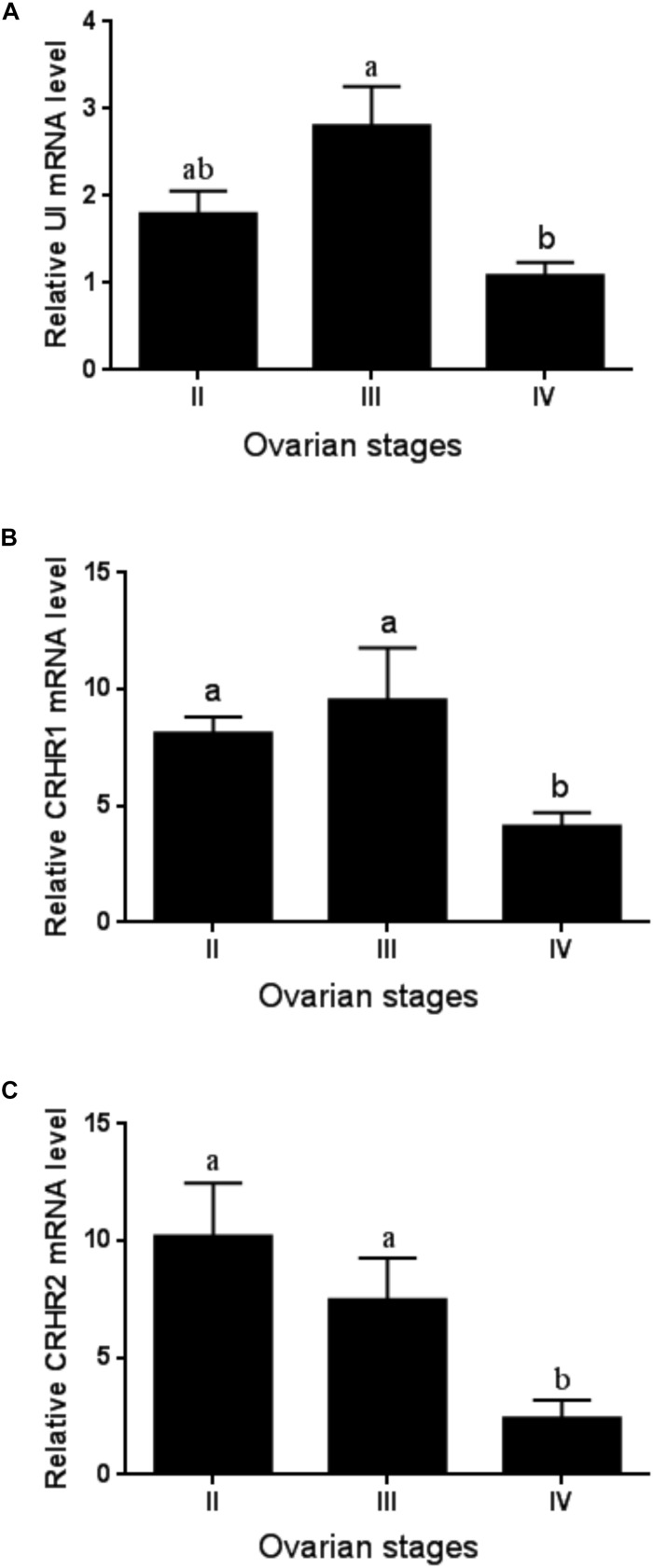
The expression of UI **(A)**, CRHR1 **(B)**, and CRHR2 **(C)** mRNA at the ovary level during ovarian development. Values are mean ± SEM, *n* = 4. Means with different letters within a column are significantly different *P* < 0.05.

The expression level of UI and CRHR1 mRNA was the highest in stage III and moderately decreased at stage IV without significant difference (Figures 2A,B). By comparison, the expression of CRHR2 mRNA levels was gradually decreased in line with the ovarian development, with the lowest in stages IV (Figure 2C). Meanwhile, the expression of the CRH receptors was much higher than that of UI gene during ovarian development.

### Stage-Dependent Expression of UI and CRH Receptors at the Follicle Level

**FIGURE 3:**
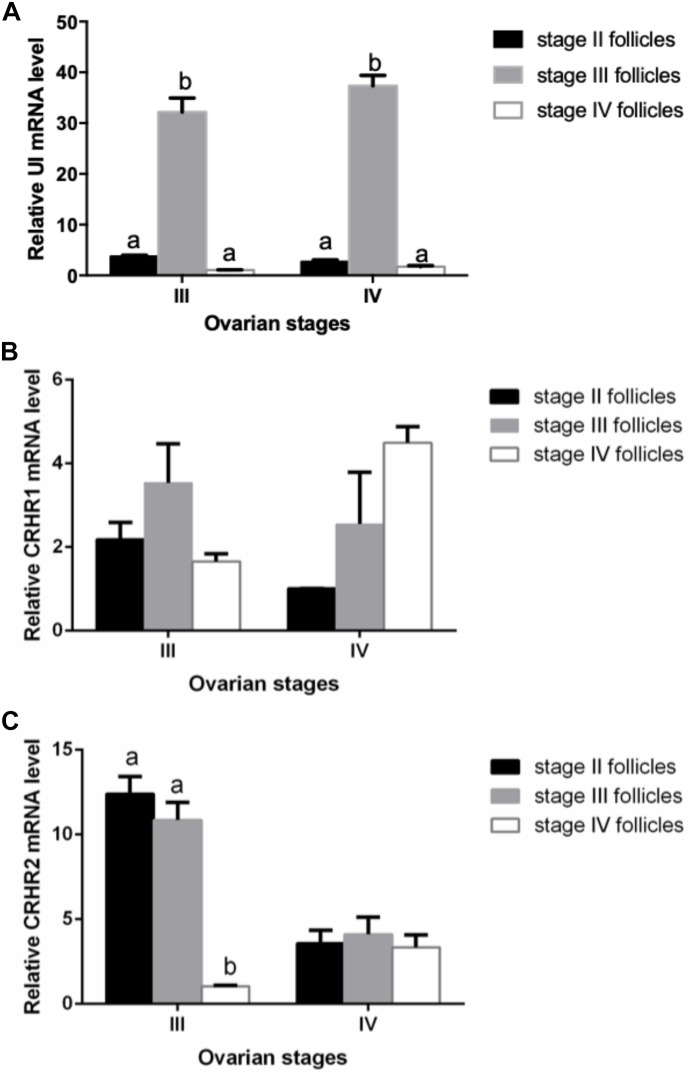
Stage-dependent expression of UI **(A)**, CRHR1 **(B)**, and CRHR2 **(C)** at the follicles in Japanese flounder. Follicles of the different stages were isolated from Japanese flounder and RNA isolated from each stage for RT-PCR analysis. The expression level was normalized to β-actin. Each data point represents the mean ± SEM of three to four replicates. Significant differences among stage of ovarian development are indicated by different letters (*P* < 0.05).

We performed experiments to detect the mRNA expression of UI and CRH receptors in stage II, III, and IV follicles for purpose of determining whether there is follicular dependence on mRNA expression of UI and CRH receptors. The expression of UI mRNA level in the stage II follicle was lower than that at the stage III follicle, increased apparently to the highest level at follicle of stage III, and then sharply decreased at stage IV follicle (Figure 3A). There was no significant difference in CRHR1 mRNA levels between the three stages of follicles (Figure 3B). The CRHR2 mRNA levels in stage IV follicles were sharply depressed compared to stage II and stage III follicle, showing a significant statistical difference (Figure 3C) (*P* < 0.05).

### Immunohistochemistry Localization of UI in the Ovarian Follicle

**FIGURE 4:**
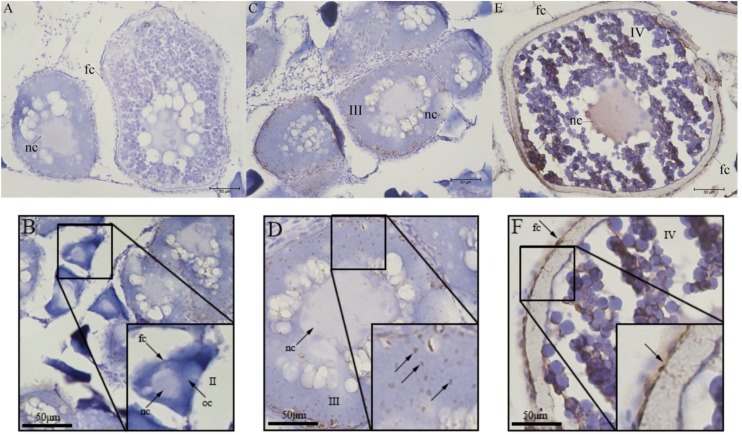
Immunohistochemical localization of UI protein in ovarian tissue of Japanese flounder. The serial sections of the ovaries were immunostained with anti-flounder UI. **(A)** Negative control, **(B)** Stage II follicles, **(C,D)** Stage III follicles, and **(E,F)** Stage IV follicles. Scale bars: 50 μm. Black arrows with/without letters represent specific locations or positive signal, respectively. Stage II 90∼300 μm, stage III 300∼500 μm, and stage IV 500∼800 μm. oc, oocyte; fc, follicular cells; nc, nucleus.

The localization of UI protein in olive flounder ovary was detected by immunohistochemistry. Immunoreactivity of UI was located at follicles and dynamic change existed in different stage follicles. Immunoreactivity of UI were detected in the stage III and stage IV oocytes and follicular cells of the ovarian follicles (Figure 4). There was no positive signal in stage II follicles (Figure 4B). In stage III follicles, immunoreactive UI was detected in cytoplasm and not found in nuclei of oocyte (Figures 4C,D). Immunoreactivity of UI was present in the follicular cells, cytoplasm and the nuclei of oocyte at the stage IV follicles (Figures 4E,F). As follicular development and vitellogenesis progress, the positive signal of UI became stronger. There was no positive signal in the negative control group sections, in which the primary antibody was replaced with PBS buffer (Figure 4A).

### Effect of hCG on the Expression of UI and CRH Receptors mRNA

**FIGURE 5:**
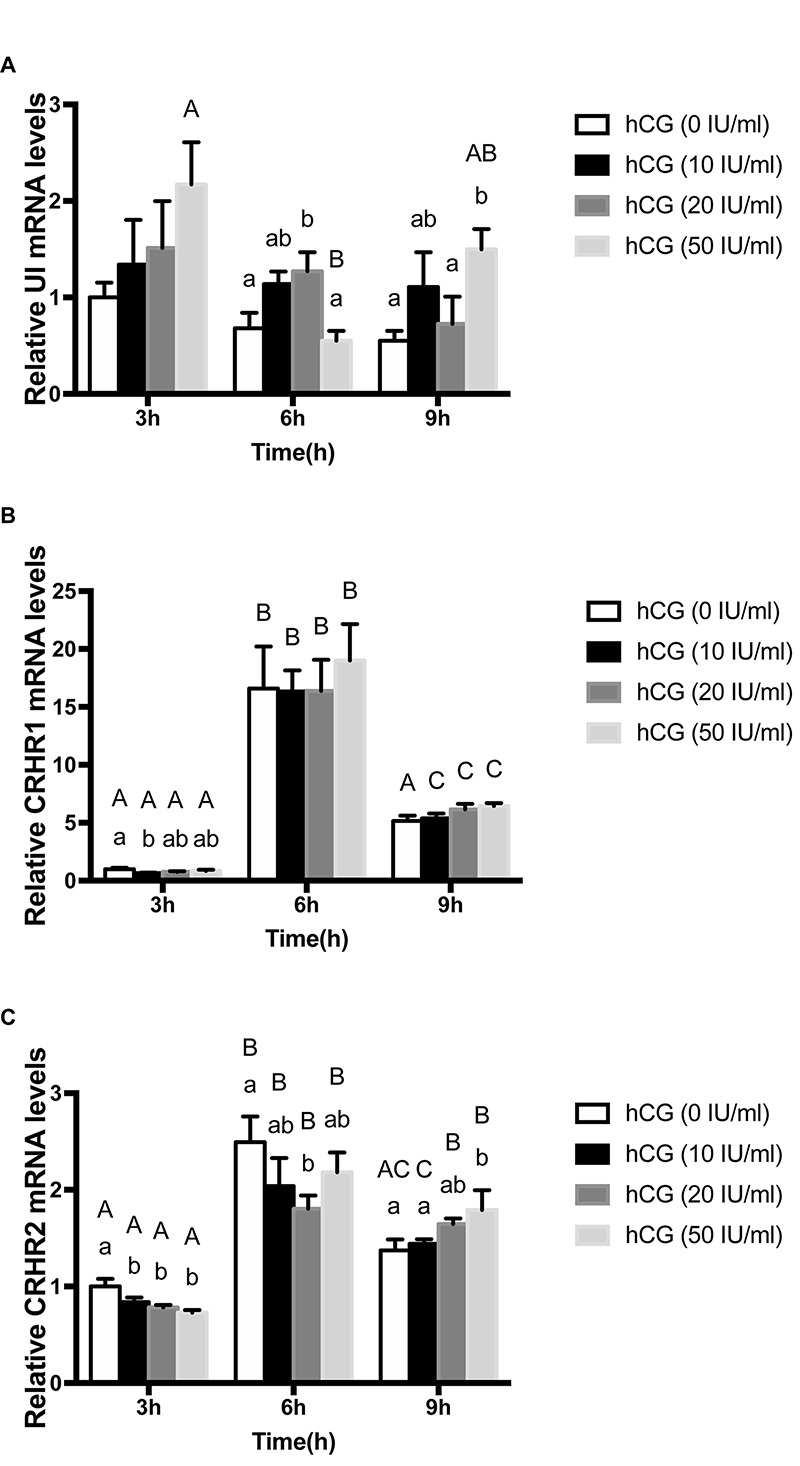
The change of expression level of UI **(A)**, CRHR1 **(B)**, and CRHR2 **(C)** mRNA in the stage IV follicles regulated by gonadotropin (hCG) *in vitro*. Data were presented as the fold change relative to hCG (0 IU/ml), which was arbitrarily set at 1. Values are means ± SEM, *n* = 6 per group. Different lowercase indicates statistical significance (*P* < 0.05) in concentration-dependent manner and different capital indicate statistical significance in time-dependent manner (*P* < 0.05).

In order to determine the effect of gonadotropins on UI and CRH receptors, the variation of UI and CRHR mRNA levels were detected in stage IV follicle by the hCG treatment *in vitro*. CRHR2 mRNA expression was markedly influenced by concentration, time and reciprocity of concentrations and time (Table 2). After three different concentration of hCG treatment, the mRNA expression of flounder UI and CRHR1 were mildly increased with no noteworthy difference compared with control group at 3 h (Figures 5A,B), but the CRHR2 mRNA levels were decreased significantly (Figure 5C). After 6 h, UI gene expression immediately increased at 10 IU/ml, reaching the highest value at 20 IU/ml. At 50 IU/ml, the UI mRNA value dropped to a lower level compared to the control group. A similar trend occurred at 9 h, but the difference is that the expression of UI remains high at 50 IU/ml (Figure 5A). After three different concentrations of hCG treatment, CRHR1 expression increased slightly compared with control group at 6 and 9 h, but this did not reach statistical significance (Figure 5B). After 6 h, the expression of CRHR2 mRNA was slightly decreased at 10 IU/ml, reaching the lowest value at 20 IU/ml and then returned to normal level at 50 IU/ml. Interestingly, this trend is contrary to the expression of the UI. The expression of CRHR2 mRNA was slightly increased at 10 IU/ml, and reached the highest level at 50 IU/ml after 9 h. Moreover, the level of CRHR2 between control group and 50 IU/ml exists remarkable differences (*P* < 0.05) (Figure 5C).

**TABLE 2:** Two-way ANOVA summary on effects of Time (T) and Concentration (C) on the expression of UI mRNA, CRHR1 mRNA, and CRHR2 mRNA.

	**UI**			**CRHR1**			**CRHR2**		
	**T**	**C**	**T^∗^C**	**T**	**C**	**T^∗^C**	**T**	**C**	**T^∗^C**
**df**	**2**	**3**	**6**	**2**	**3**	**6**	**2**	**3**	**6**
MS	1.503	0.973	0.501	1608.759	6.098	2.911	9.630	0.180	0.304
F	4.375	2.831	1.458	91.022	0.345	0.165	1.488	79.669	2.511
P	0.020	0.053	0.222	<0.001	0.793	0.985	<0.001	0.228	0.032

*Time, 3, 6, and 9 h; Concentration, 0, 10, 20, and 50 IU/ml hCG.*

### Effect of DHP on the Expression of UI and CRH Receptors mRNA

**FIGURE 6:**
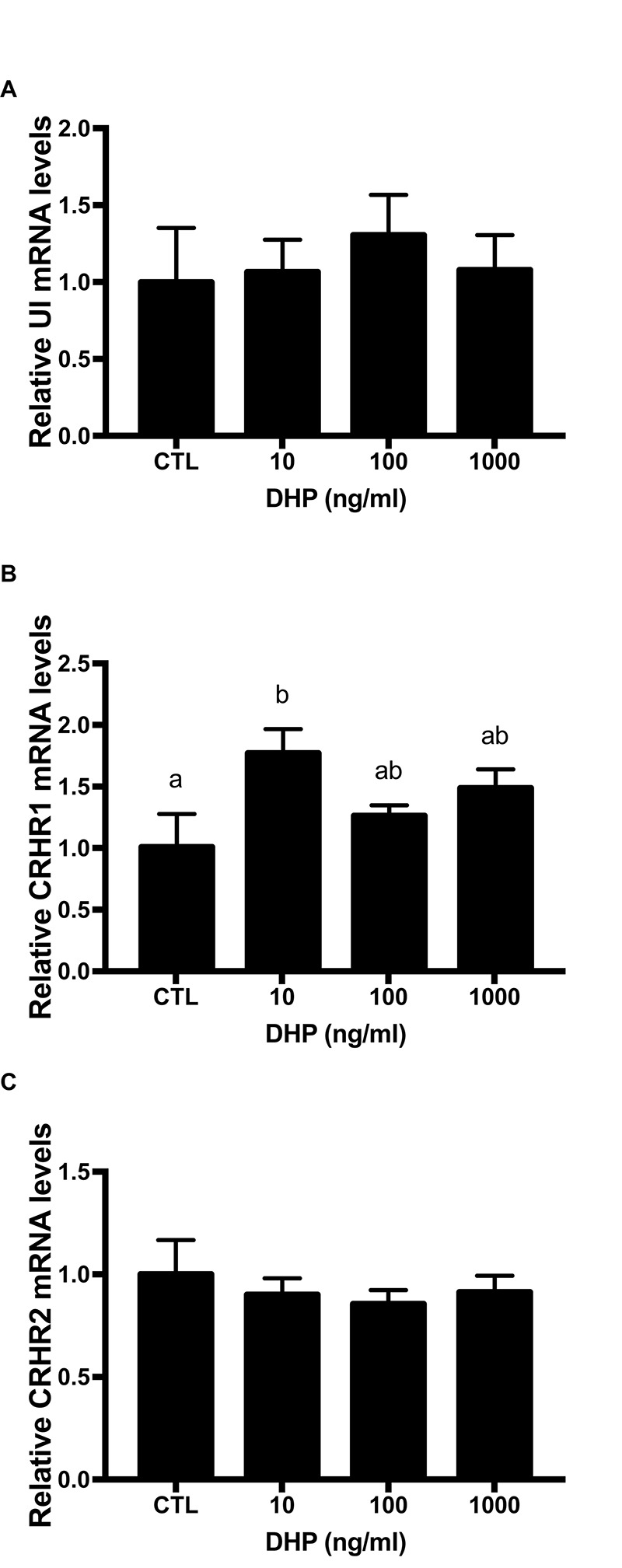
mRNA expression for UI **(A)**, CRHR1 **(B)**, and CRHR2 **(C)** mRNA in the stage IV follicles induced by DHP for 9 h *in vitro*. Data were presented as the fold change relative to control group, which was arbitrarily set at 1. Values are means ± SEM, *n* = 6 per group. Means with different letters within a column are significantly different *P* < 0.05.

UI gene expression immediately increased at 10 ng/ml, reaching the highest value at 100 ng/ml. At 1000 ng/g, the UI value dropped to a higher level compared to the control group. But, there was no significant difference on UI level after 9 h DHP treatment (Figure 6A). Similarly, after three different concentration of DHP treatment, flounder CRHR1 expression was gradually elevated compared with control group at 9 h. In addition, there was a noteworthy discrepancy between 10 ng/ml and control group (*P* < 0.05) (Figure 6B). However, after three different concentration of DHP treatment, CRHR2 expression in flounder decreased slightly compared with control group at 9 h (Figure 6C).

## Discussion

Urotensin I was considered to play an important role in osmotic regulation, vasoconstriction, suppression of orexis, locomotion, cortisol release, and immunes response in teleost (Singh and Rai, 2011). In additional, there are some reports that Ucn I also perform functions in reproduction system in mammals. The levels of Ucn I mRNA in the regressing corpus luteum were significantly higher than that of functioning corpus luteum in normal human ovaries (Muramatsu et al., 2001). The expression of Ucn I gene in granulosa cells reached its maximum in the mid-luteal phase and decreased in the late luteal phase (Xu et al., 2006, 2007). The dynamic levels of UI, CRHR1 and CRHR2 mRNA were firstly detected in the olive flounder ovary in our study. Interestingly, the mRNA expression of UI, CRHR1, and CRHR2 in the ovary were high at the stage III, but sharply decreased at the stage IV. Meanwhile, UI protein could be detected in the follicular cells and oocytes in stage III and IV follicles of olive flounder. The expression of UI and CRH receptors were regulated by hCG and DHP.

The mRNA expression of urocortin and CRH receptors was detected in the human and rhesus macaque ovary, and CRH system is involved in the regulation of mammalian reproduction (Xu et al., 2006; Zoumakis et al., 2009; Makrigiannakis et al., 2018). High sequence similarity between mammal and teleost suggested a conserved evolutionary role among CRH-like peptide in vertebrates. Our previous study has demonstrated expression levels of mRNA for CRH-related peptides, CRH and UI, shown seasonal changes that reached its lowest expression in April and maximum in August to October in CNSS of euryhaline flounder *Platichthys flesus* (Lu et al., 2007). The CNSS which is the major contributor for circulating UI was functionally reprogrammed to cope with physiological changes that was used for winter body maintenance and gonadal development (Lu et al., 2007). Combined with the current study, the dynamic expression of UI mRNA in follicles at different developmental stages of different ovarian developing stages, the ovarian UI, and CNSS UI all contribute to gonadal development.

The CNSS have been well accepted to be the major site where UI were produced, then released by urophysis (Lu et al., 2004). Previous study shown mature breeders of *Liza ramada* might enhance the synthesis and/or secretion of CRH immunoreactive cells in the nucleus preopticus (NPO) and medulla oblongata (MO) and respond to stress resulting from ovarian maturation and spawning (Mousa and Mousa, 2006). In human ovary, Ucn I with weak immunoreactivity was discovered in thecal internal cells of dominant/non-dominant follicles (including follicles and luteal phase), as well as in granulosa cells of dominant and atresia follicles (Muramatsu et al., 2001). Ucn I could not be detected in rhesus macaque ovaries during the preovulatory phase of corpus luteum (Xu et al., 2006). During the follicular phase, it was reported that CRH, another CRH-related peptide, was found in both theca cells of growing antral (7–8 mm in diameter) and the dominant follicles in the human ovary (Muramatsu et al., 2001; Xu et al., 2006). Our study is the first report to provide the detailed localization of UI in the ovary during ovarian development in olive flounder. Immunoreactive UI were detected in the follicular cells and oocyte at the stage III follicle in olive flounder ovary. These results suggested the expression and function of CRH-related peptide maybe stage-specific or species-specific.

On the contrary, in the mid- and late-corpus luteum, immunoreactive Ucn I was discovered in both thecal cells and luteinized granulosa after ovulation (luteal phase). During early corpus luteal degeneration, luteinized thecal cells, but not in luteinized granulosa cells and the *Corpus albicans*, showed strong Ucn I immunoreactivity. Ultimately, the immunoreactivity of luteinized granulosa and thecal cells to Ucn I was weak in the corpus luteum from pregnant females (Muramatsu et al., 2001; Florio et al., 2004). Therefore, Ucn I have been presumed to be synthesized locally from steroidogenic luteal cells and to function in autocrine and/or paracrine ways (Wypior et al., 2011). Our immunohistochemistry results indicated that UI proteins were associated with the follicular cells and oocytes during the ovarian development. In stage II follicle, immunoreactive UI was not detected in both follicular cells and oocyte. However, in the stage III and stage IV, a strong signal of immunoreactivity for UI has been detected, suggested that processed causing or associated with postvitellogenic follicles. It implied that UI may not be involved in previtellogenesis but in the postvitellogenesis during the ovarian development of flounder. A recent study in Japanese eel *Anguilla japonica* has shown that GnRH neurons and CRH neurons exist reciprocal connections in the brain (Amano et al., 2014). Whether ovarian UI has been involved in teleost oocyte maturation and/or ovulation will be an interesting problem to be solved in the future.

Although some researches have shown the expression and secretion of CRH-related peptides might regulated by hormones, but the details of the regulatory mechanisms of UI secretion from peripheral tissues were unknown. Gonadotropin and DHP were known to play major roles in ovarian development and oocyte maturation in teleost. In common carp *Cyprinus carpio*, the DHP content in the plasma elevated significantly in specimens with migrating oocytes compared to other stages (Aizen et al., 2012; Vazirzadeh et al., 2014). In greater amberjack *Seriola dumerili*, the concentration of plasma LH slowly elevated from cortical alveolus and kept high level in middle vitellogenesis and atresia (Nyuji et al., 2016). Studies have shown that hCG induces the maturation of oocyte by interacting with fish receptors of LH in a similar manner to fish luteinizing hormone (LH) (Tan et al., 2009). CRH immunoreactive cells in the NPO and MO dramatically increased their secretory activity during spawning induced by hCG (Mousa and Mousa, 2006). In primates, gonadotropin has no effect on the expression of CRH receptors (Xu et al., 2006). The expression of Ucn I has no obvious difference when treated with the antagonists or agonists of gonadotropin-releasing hormone (Sanchez et al., 2010; Celik et al., 2013). In the current study, the gonadotropin altered UI and CRH receptors expression in isolated ovarian follicles in time-dependence manner. Although there was no remarkable change in the mRNA expression of UI and CRHR2 after 9 h of incubation with DHP, however, DHP could induce expression changes of CRHR1. Together, these studies indicated that hCG or DHP were involved in the regulation of UI and CRH receptors expression in the ovary of the olive flounder. Studies on the effects of UI and CRH receptors on reproduction are rare in teleost, so further studies are needed to elucidate how CRH-related peptides regulate the reproduction of olive flounder.

## Conclusion

To sum up, our research provided the first particular analysis of the UI and CRH receptors expression in teleost during ovarian development. The profile of UI and CRHR mRNA expression and localization of UI in the ovary and follicle level suggested that UI might involve in postvitellogensis during ovarian development in teleost. Furthermore, the expression of UI and CRH receptors in the follicle might regulated by the hCG or DHP. These results indicated that UI and CRH receptors may play an important role in olive flounder ovarian development.

## Data Availability

All datasets generated for this study are included in the manuscript and/or the supplementary files.

## Ethics Statement

The experimental program was approved by the Animal Ethics Committee of Shanghai Ocean University (Shanghai, China) and complied with the Guidelines on Ethical Treatment of Experimental Animals set by the Ministry of Science and Technology, China.

## Author Contributions

HZ, AC, and WL designed the experiments and wrote the manuscript. HZ and CG carried out the experiments. HZ analyzed the experimental results.

## Conflict of Interest Statement

The authors declare that the research was conducted in the absence of any commercial or financial relationships that could be construed as a potential conflict of interest.
